# Signaling pathways in human osteoclasts differentiation: ERK1/2 as a key player

**DOI:** 10.1007/s11033-020-06128-5

**Published:** 2021-01-24

**Authors:** Paula Pennanen, Roope A. Kallionpää, Sirkku Peltonen, Liisa Nissinen, Veli-Matti Kähäri, Eetu Heervä, Juha Peltonen

**Affiliations:** 1 Department of Cell Biology and Anatomy, Institute of Biomedicine, University of Turku, Kiinamyllynkatu 10, 20520 Turku, Finland; 2 Department of Dermatology, University of Turku and Turku University Hospital, Turku, Finland; 3 Department of Dermatology and Venereology, Institute of Clinical Sciences, Sahlgrenska Academy, University of Gothenburg, Gothenburg, Sweden; 4 Department of Dermatology and Venereology, Region Västra Götaland, Sahlgrenska University Hospital, Gothenburg, Sweden; 5 MediCity Research Laboratory, University of Turku and Cancer Research Laboratory FICAN West, University of Turku and Turku University Hospital, Turku, Finland; 6 Department of Oncology, University of Turku and Turku University Hospital, Turku, Finland

**Keywords:** Osteoclast, Neurofibromatosis 1, Signaling pathways, p38, ERK1/2

## Abstract

Little is known about the signaling pathways involved in the differentiation of human osteoclasts. The present study evaluated the roles of the Ras/PI3K/Akt/mTOR, Ras/Raf/MEK1/2/ERK1/2, calcium-PKC, and p38 signaling pathways in human osteoclast differentiation. Mononuclear cells were isolated from the peripheral blood of control persons and patients with neurofibromatosis 1 (NF1), and the cells were differentiated into osteoclasts in the presence of signaling pathway inhibitors. Osteoclast differentiation was assessed using tartrate-resistant acid phosphatase 5B. Inhibition of most signaling pathways with chemical inhibitors decreased the number of human osteoclasts and disrupted F-actin ring formation, while the inhibition of p38 resulted in an increased number of osteoclasts, which is a finding contradictory to previous murine studies. However, the p38 inhibition did not increase the bone resorption capacity of the cells. Ras-inhibitor FTS increased osteoclastogenesis in samples from control persons, but an inhibitory effect was observed in NF1 samples. Inhibition of MEK, PI3K, and mTOR reduced markedly the number of NF1-deficient osteoclasts, but no effect was observed in control samples. Western blot analyses showed that the changes in the phosphorylation of ERK1/2 correlated with the number of osteoclasts. Our results highlight the fact that osteoclastogenesis is regulated by multiple interacting signaling pathways and emphasize that murine and human findings related to osteoclastogenesis are not necessarily equivalent.

## Introduction

**Fig. 1:**
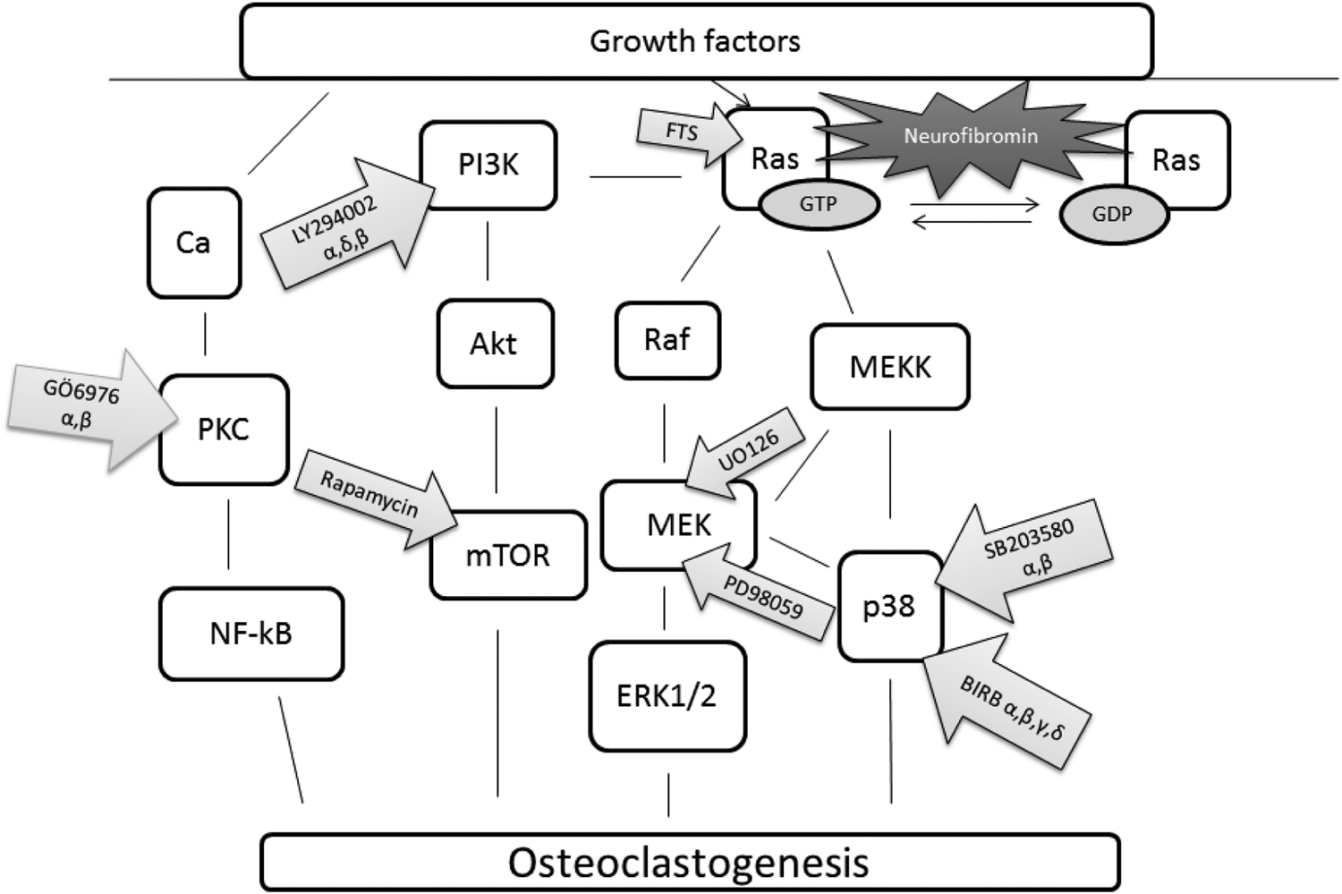
Schematic presentation of cell signaling pathways and inhibitors used in this study. Eight inhibitors were utilized to explore the p38, Ras/PI3K/Akt/mTOR, Ras/Raf/MEK/ERK1/2, and calcium-PKC signaling pathways of human osteoclast differentiation and function

Osteoclast differentiation from the cells of the monocyte-macrophage lineage is regulated by receptor activator of nuclear factor kappa-B ligand (RANKL) and macrophage colony-stimulating factor (M-CSF) [1, 2]. Several signaling pathways including p38, Raf / MEK1/2 / ERK1/2, calcium-PKC, and PI3K / Akt / mTOR are mediated by Ras and are all involved in the differentiation and function of osteoclasts (Fig. 1) [3]. These pathways have mostly been studied using cultured rodent osteoclasts exposed to inhibitors of the different signaling pathways one at a time. The emergence of targeted signaling pathway inhibitors in clinical use especially in oncology highlights the need to better understand the effects of these pathways in human osteoclast differentiation.

The p38 can be blocked by BIRB796, a potent inhibitor of all p38 isoforms. Another inhibitor of p38, SB203580, is selective for the α and β isoforms of p38 [4, 5]. Inhibition of p38 with SB203580 decreases the number of murine osteoclasts [6, 7] and blocks the effect of external stimulation of osteoclastogenesis [8]. Activation of p38 in mice is in turn associated with enhanced survival of osteoclasts [9].

The inhibition of the Raf/MEK-pathway is largely reflected by the phosphorylation of ERK1/2 [10]. U0126 and PD98059 prevent the activation of MEK1/2 and inhibit activated MEK1/2. Conflicting results have been reported on the effect of MEK inhibitors on murine osteoclast differentiation [7, 11]. However, it seems that MEK inhibition can suppress osteoclast differentiation in murine samples [12, 13]. Inhibition of calcium-PKC with GÖ6976 decreases the phosphorylation of ERK1/2 and the number of murine osteoclasts [14, 15].

M-CSF and RANKL enhance the proliferation of osteoclast precursors, survival, and activity of osteoclasts through the phosphoinositide 3-kinase (PI3K) / protein kinase B (Akt) pathway [3]. The inhibition of PI3K with LY294002 has been shown to suppress differentiation and survival of rodent osteoclasts [16–18]. The inhibition of the mammalian target of rapamycin (mTOR) is known to decrease the growth of long bones in rodents and is thus associated with the downregulation of bone formation [19]. Several studies have reported that inhibition of mTOR with rapamycin decreases osteoclast differentiation of rodent cells [20–22].

Downstream pathways influenced by Ras may activate or suppress osteoclast resorption [16]. Farnesyl thiosalicylic acid (FTS) inhibits all isoforms of Ras [23]. A study using mouse osteoclasts showed that inhibition of Ras decreased osteoclast survival [16]. Another study found that the inhibition of Ras with FTS decreases the number of mononuclear cells in rats, yet the effects on osteoclastogenesis were not assessed [24].

The *NF1* gene encodes the tumor suppressor protein neurofibromin, which is a negative regulator of Ras. Germline *NF1* mutations cause the autosomal dominant neuro-cutaneous-skeletal syndrome neurofibromatosis type 1 (NF1), which has an incidence of 1/2000 [25–27]. Neurofibromin is needed for normal bone development [28] and mutations in the *NF1* gene may cause skeletal abnormalities. Bone lesions in NF1 are variable and include scoliosis, short stature, osteopenia, and osteoporosis [29, 30]. A gain-in-function of osteoclasts leading to increased bone resorption has been demonstrated in cells from NF1 patients [31, 32]. NF1 osteoclasts are also more resistant to apoptosis compared to healthy control osteoclasts [31].

This study focuses on the roles of different signaling pathways in the differentiation of human osteoclasts. Osteoclasts from control persons and patients with NF1 were compared to model genetically up-regulated Ras-signaling in osteoclast differentiation.

## Materials and methods

### Ethical approval, participants and informed consent

The study was carried out at the Turku University Hospital and the University of Turku, Finland. It complied with the Declaration of Helsinki, was approved by the Ethics Committee of Southwest Finland Hospital District, and had research permission granted by the Turku University Hospital. The participants gave their written informed consent for establishing osteoclast cultures from their peripheral blood samples. The participants did not use any medication known to affect bone, and they were aged 18–62 years (median age was 31 years). NF1 patients fulfilled the NIH diagnostic criteria [33].

### Cell culture and differentiation of human osteoclasts on glass coverslips

Mononuclear cells including osteoclast precursors were isolated with Ficoll-Paque PLUS (GE Healthcare Bio-Sciences, Uppsala, Sweden) centrifugation from fresh blood samples of 7 healthy controls (4 males, 3 females) and 8 NF1 patients (5 males, 3 females), aged 18–59 years (median age was 27 years), using the protocol described by Husheem et al. [34] and modified by Pennanen et al. [35]. Half a million mononuclear cells were seeded per glass coverslip (12 mm round coverslips with thickness of 0.17 mm H = 1.5, Marienfeld GmbH &Co.KG, Lauda-Königshofen, Germany) and differentiated into multinuclear osteoclasts in medium containing alpha-MEM (Gibco, Grand Island, NY, USA), 10% heat-inactivated fetal bovine serum (Gibco), penicillin-streptomycin (Lonza, Basel, Switzerland), RANKL (20 ng/ml, Peprotech, Rocky Hill, NJ, USA), and M-CSF (10 ng/ml, Peprotech) for 4–5 days. The inhibitors (Table 1) were present in the culture medium throughout the culturing. For each person and inhibitor, three to four parallel cultures were established.

**Table 1:** Summary of inhibitors

Inhibitor	Manufacturer	Target	Concentration in culture medium
Vehicle +DMSO	–	Control	0.05%
FTS	Cayman chemical company, Ann Arbor, MI, USA	all isoforms of Ras	10 μM
GÖ6976	Calbiochem, San Diego, CA, USA	PKC α, β	0.01 μM
U0126	Cell Signaling, Danvers, MA, USA	MEK	0.1 μM
PD98059	Calbiochem, San Diego, CA, USA	MEK	1 μM
LY294002	Cell Signaling, Danvers, MA, USA	PI3K α, δ, β	1 μM
Rapamycin	Cell Signaling, Danvers, MA, USA	mTOR	0.001 μM
BIRB796	Axon Medchem BV, Groningen, the Netherlands	p38 α, β, γ, δ	10 μM
SB203580	Calbiochem, San Diego, CA, USA	p38 α, β	1 μM

### Assessment of osteoclast differentiation with TRACP-staining

The differentiation of osteoclasts was assessed by analyzing tartrate-resistant acid phosphatase form 5b (TRACP), which is a commonly approved cytochemical marker of osteoclast activity [36]. The differentiation of mononuclear cells into osteoclasts on glass coverslips was assessed after 4–5 days of culture, which was the time required for the formation of identifiable differentiated osteoclasts. The cells were fixed with 4% paraformaldehyde and stained for TRACP (Sigma-Aldrich leukocyte acid phosphatase-kit, Steinheim, Germany) according to manufacturer’s instructions. To visualize the nuclei, cells were stained with Hoechst 33342 (Invitrogen, Eugene, OR, USA; catalogue number H3570) in 1:10000 dilution in 1% BSA in PBS for one hour at room temperature. The cells were imaged with a Carl Zeiss Axioimager microscope and Carl Zeiss Zen 2012 (blue edition, version 1.1.2.0) software using 20x magnification. The area with the highest cell density was always chosen for imaging, and the number of osteoclasts was counted from four microscopic fields. TRACP-positive cells with three or more nuclei were considered to be osteoclasts. To assess osteoclast size in SB203580, BIRB796 and vehicle treated experiments, 20 largest osteoclasts were visually identified in a tiled image of 25 microscopic fields and the largest diameter of each of these osteoclasts was measured using the QuPath software, version 0.1.2 [37].

### The quantification of osteoclast bone resorption activity

In a separate experiment, osteoclast progenitors of three control persons and three NF1 patients matched by age and gender were differentiated into osteoclasts essentially as described above, but on bovine bone slices (purchased from Pharmatest Services Ltd., Turku, Finland). Half a million cells were seeded per bone slice on a 96-well-plate and cultured in the presence of the inhibitors listed in Table 1 or a vehicle control for 16 days. Half of the medium was replaced with fresh medium every 3–4 days.

The carboxy-terminal cross-linking telopeptide of type I collagen (CTX) in cell culture media was used as an indicator of the bone resorption activity of the osteoclasts [36]. Culture media from five parallel experiments of osteoclasts cultured on bone slices with the p38 inhibitor SB203580 or vehicle were used. The CTX analysis was carried out after 16 days of culture in order to allow enough time for full differentiation of cells from all donors. CTX assays were purchased from Pharmatest Services Ltd., Turku, Finland.

### Fluorescent staining of osteoclasts to assess F-actin ring formation

The effects of the different inhibitor treatments on osteoclast differentiation on bovine bone slices were studied by fluorescent staining of nuclei and F-actin rings. First, osteoclasts were fixed with 4% paraformaldehyde, permeabilized with 0.01% Tween-20 in PBS on ice for 5 min, and non-specific binding was blocked with 1% BSA in PBS for 30 min. Cells were stained with STAR635 phalloidin (Abberior GmbH, Gottingen, Germany; catalogue number 2-0205-002-5) in 1:100 dilution to visualize F-actin and with Hoechst 33342 at a 1:10000 dilution in 1% BSA in PBS to visualize the nuclei, both for 1 h at room temperature. Glass coverslips were embedded with 30% glycerol in PBS and imaged with Carl Zeiss Axioimager microscope and Carl Zeiss Zen 2012 (blue edition) software.

### Western blot analyses

Osteoclasts from three healthy donors and three NF1 patients matched by age and gender were cultured in 24-well plates for 5 days for western blot analysis. The cell lysates were collected by scraping in Laemmli sample buffer supplemented with Phosphatase Inhibitor Cocktail II (Sigma-Aldrich, St. Louis, MO, USA) and Complete Mini Protease Inhibitor Cocktail (Roche Diagnostics, Mannheim, Germany), and lysates from three parallel wells were pooled. The lysates were denatured for 5 min at 95 °C, run in 8% SDS-PAGE gels, and western blotted onto polyvinyl difluoride membranes (EMD Millipore, Billerica, MA, USA). The membranes were blocked with 5% milk. The membranes were incubated with primary antibodies for phospho-p38 MAPK (Thr180/Tyr182) (1:1000, Cell Signaling Technology, Danvers, MA, USA; catalogue number #9211) and total p38 MAPK (1:1000, Cell Signaling Technology #9212) in 5% BSA / TBS-Tween-20, and for phospho-Akt (Thr308) (1:1000, Cell Signaling Technology #13038), and pan-Akt (1:1000, Cell Signaling Technology #4691) in 5% milk / TBS-Tween-20 overnight at 4 °C. The incubations with primary antibodies for phospho-p44/42 MAPK (ERK1/2, Thr202/Tyr204) (1:1000, Cell Signaling Technology #4377) and total p44/42 MAPK (ERK1/2) (1:1000, Cell Signaling Technology #4695) were in 1% BSA/PBS-Tween-20 at room temperature for 1 h. The primary antibodies were followed by washing and incubation with horseradish peroxidase-linked goat anti-rabbit antibody (1:1000, Cell Signaling Technology #7074) at room temperature for 30 min. The antibodies were detected by chemiluminescence and imaged with a LAS-4000 device (Fujifilm, Tokyo, Japan). For each protein, the phosphorylated and total protein were always assayed from the same membrane. Detection of the phosphorylated form was followed by stripping of the membrane in a solution containing 2% SDS, 0.8% β-mercaptoethanol, and 65 mM Tris-HCl pH 6.8 at 55 °C for 30 min.

The resulting blots were analysed by subtracting background, and each band was quantified using the built-in tools of the ImageJ software (version 1.49 m; imagej.nih.gov/ij/). The ratios of phosphorylated-to-total protein were computed for each band. Thus, the amounts of total p38, Akt and ERK1/2 served as loading controls for the respective phosphorylated proteins. Data from each donor’s cells were normalized relative to the vehicle control.

### Statistics

Comparisons of cell counts between inhibitors and between healthy and NF1 were performed using generalized linear mixed effects models with Poisson distribution and nested random intercepts for matched pairs and each person. The diameter of osteoclasts was compared between treatments using square root transformed values and linear mixed effects models with a random intercept for each person. In the CTX assay, the wells with the highest and lowest readings from each donor were excluded to avoid any erroneous values, leaving data from three parallel assays. Statistical analyses and visualization were performed using the R software version 3.3.0 (www.r-project.org) and packages lmerTest (2.0–32) and lme4 (version 1.1–12). *P*-values <0.05 were considered as significant.

## Results

### The effects of inhibitors of PKC, MEK, PI3K, and mTOR on osteoclast differentiation

**Fig. 2:**
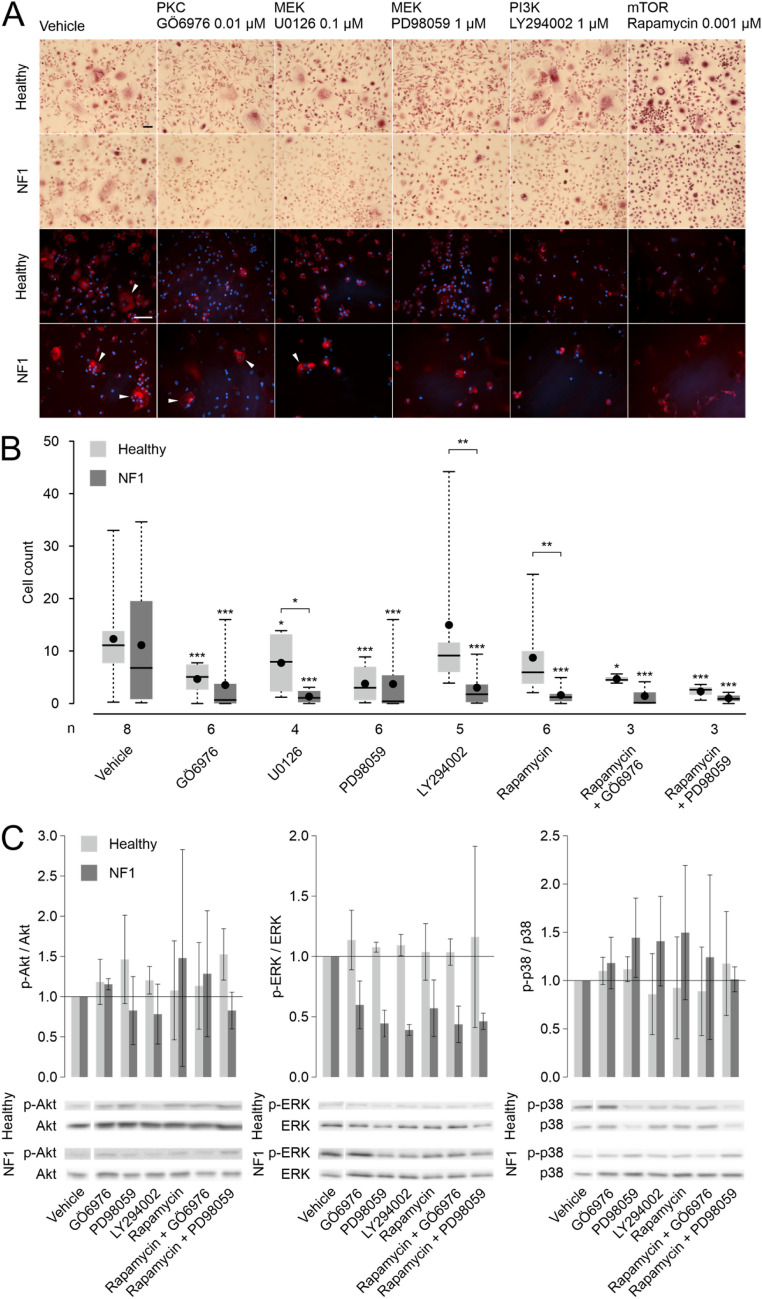
The effects of inhibition of PKC, MEK, PI3K, and mTOR on osteoclast differentiation. (**a**) The first two rows show the tartrate-resistant acid phosphatase staining of cells isolated from the peripheral blood of control persons and patients with neurofibromatosis type 1 (NF1) and cultured with inhibitors of PKC (GÖ6976), MEK (PD98059 and U0126), PI3K (LY294002) and mTOR (Rapamycin) on glass. The two bottom rows show actin staining (red) on bone together with nuclei (blue). White arrows indicate examples of osteoclasts in the fluorescence images. Scale bars are 50 μm. (**b**) Counts of osteoclasts after 4–5 days of differentiation in the presence of selected signaling pathway inhibitors. The thick horizontal line represents the median, the black dot shows the mean, the box displays the 1st and 3rd quartiles, and the whiskers show the range of values. The number (n) of matched healthy-NF1 pairs is shown for each treatment. Asterisks denote significance of difference to vehicle control (above each box) or between healthy and NF1. *, 0.01 < *P* < 0.05; **, 0.001 < *P* < 0.01; ***, *P* < 0.001. (**c**) Effects of the signaling inhibitors on the phosphorylation of Akt (60 kDa), ERK1/2 (42/44 kDa), and p38 (41 kDa). The bars show mean ± standard deviation of phosphorylated protein / total protein normalized relative to the vehicle treatment. The data are representative of three experiments with cells from different individuals. Examples of bands observed by western blotting are shown below each figure. Since the samples shown in Figs. 2, 3 and 4 were run on the same gel, the vehicle control band for each antibody is shared by the figures. (Color figure online)

Visual inspection of the cultures treated with inhibitors of PKC (GÖ6976), MEK (PD98059 or U0126), PI3K (LY294002), and mTOR (Rapamycin) revealed viable, normal looking cells on the entire substratum (Fig. 2a). However, visualization of F-actin with phalloidin staining revealed that the inhibitors prevented the formation of typical F-actin rings in healthy and NF1 osteoclasts.

The formation of osteoclasts under vehicle treatment did not differ between mononuclear cells from healthy donors and NF1 patients. The number of osteoclasts decreased, when mononuclear cells from healthy individuals were treated with inhibitors of PKC or MEK (Fig. 2b). Inhibition of PI3K or mTOR did not significantly affect the number of osteoclasts from healthy donors. When osteoclasts derived from NF1 patients were treated with inhibitors of PKC, MEK, PI3K, or mTOR, the number of osteoclasts decreased significantly in all cases (Fig. 2b). The combination of inhibitors of mTOR and MEK decreased the number of osteoclasts even more potently in both healthy and NF1 cells as compared to MEK inhibition only (*P* = 0.010 and *P* < 0.001, respectively). Adding an inhibitor of mTOR enhanced the effect of PKC inhibition only in NF1 cells (*P* < 0.001) (Fig. 2b).

Western blotting showed that the inhibition of PKC, MEK, PI3K, and mTOR had no major effect on the phosphorylation of ERK1/2 or p38 in healthy cells. However, in NF1 cells, decreased phosphorylation of ERK1/2 was observed with all of the used inhibitors (Fig. 2c). This coincides with the lower number of osteoclasts in the inhibitor-treated NF1 samples (Fig. 2b). In addition, in NF1 cells, the phosphorylation of p38 was increased by inhibition of MEK, PI3K, and mTOR (Fig. 2c). Inhibition of PKC, PI3K, or mTOR showed no marked effect on the phosphorylation of Akt, yet inhibition of MEK alone or in combination with mTOR increased the phosphorylation of Akt in cells from healthy donors (Fig. 2c).

### The effects of inhibition of p38 with SB203580 and BIRB796 on osteoclast differentiation

**Fig. 3:**
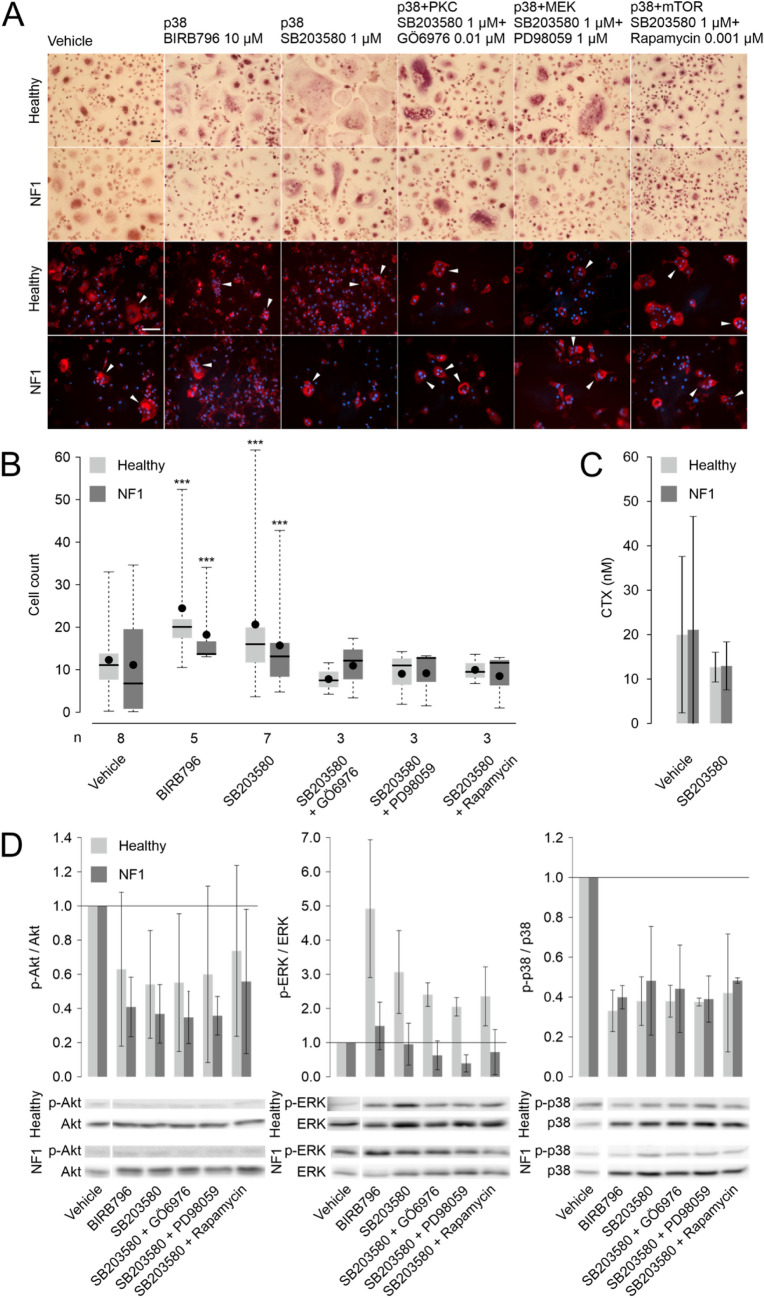
The effects of p38 inhibition by SB203580 and BIRB796 on osteoclast differentiation. (**a**) The two uppermost panels show the tartrate-resistant acid phosphatase staining of cells isolated from the peripheral blood of control persons and patients with neurofibromatosis type 1 (NF1) cultured on glass in the presence of inhibitors of p38. The two bottom rows show actin staining (red) on bone together with nuclei (blue). White arrows indicate examples of osteoclasts in the fluorescence images. Scale bars are 50 μm. (**b**) Counts of osteoclasts after 4–5 days of differentiation in the presence of selected signaling pathway inhibitors. The thick horizontal line represents the median, the black dot shows the mean, the box displays the 1st and 3rd quartiles, and the whiskers show the range of values. The number (n) of matched healthy-NF1 pairs is shown for each treatment. Asterisks denote significance of difference to vehicle control (above each box) or between healthy and NF1. *, 0.01 < *P* < 0.05; **, 0.001 < *P* < 0.01; ***, *P* < 0.001. (**c**) The effect of p38 inhibition on the concentration of C-terminal telopeptide (CTX) measured from cell culture medium after 16 days of culture. The bars show mean ± standard deviation after exclusion of the wells with the highest and lowest readings from each donor. (**d**) Effects of the signaling inhibitors on the phosphorylation of Akt (60 kDa), ERK1/2 (42/44 kDa), and p38 (41 kDa). The bars show mean ± standard deviation of phosphorylated protein / total protein normalized relative to the vehicle treatment. The data are representative of three experiments with cells from different individuals. Examples of bands observed by western blotting are shown below each figure. Since the samples shown in Figs. 2, 3 and 4 were run on the same gel, the vehicle control band for each antibody is shared by the figures. (Color figure online)

Inhibition of p38 resulted in marked changes in the morphology of osteoclasts (Fig. 3a), and the number of osteoclasts increased significantly when control persons and NF1 patients’ cells were treated with the p38 inhibitors SB203580 or BIRB796 (Fig. 3b). Specifically, the inhibition of p38 led to osteoclasts larger in size compared to cells cultured without inhibitor in both healthy and NF1 cells. In healthy cells, the average diameter of the largest osteoclasts was 1.87-fold in experiments treated with BIRB796 and 1.63-fold in experiments treated with SB203580 compared to vehicle control (*P* < 0.001). The respective numbers were 1.81 and 1.57 in NF1 cells (*P* < 0.001). The osteoclasts displayed undisturbed F-actin ring formation. However, the p38 inhibition did not increase the level of CTX in the culture media indicating stable resorption activity (Fig. 3c). Western blot analyses confirmed that inhibition of p38 decreased the ratio of phosphorylated and total p38 by 52–67% in healthy and NF1 cells (Fig. 3d). The inhibition of p38 was associated with an increase in ERK1/2 phosphorylation observed in control but not in NF1 cells, while a decrease in Akt phosphorylation was observed in both control and NF1 cells (Fig. 3d).

Increased osteoclastogenesis induced by p38 inhibition was counteracted by the inhibitors of PKC, MEK, and mTOR (Fig. 3b). In combined inhibitor treatments, osteoclast numbers were at the vehicle level. Akt and p38 phosphorylation levels remained low in combined inhibitor treatments (Fig. 3d).

### The effects of Ras inhibition on osteoclast differentiation

**Fig. 4:**
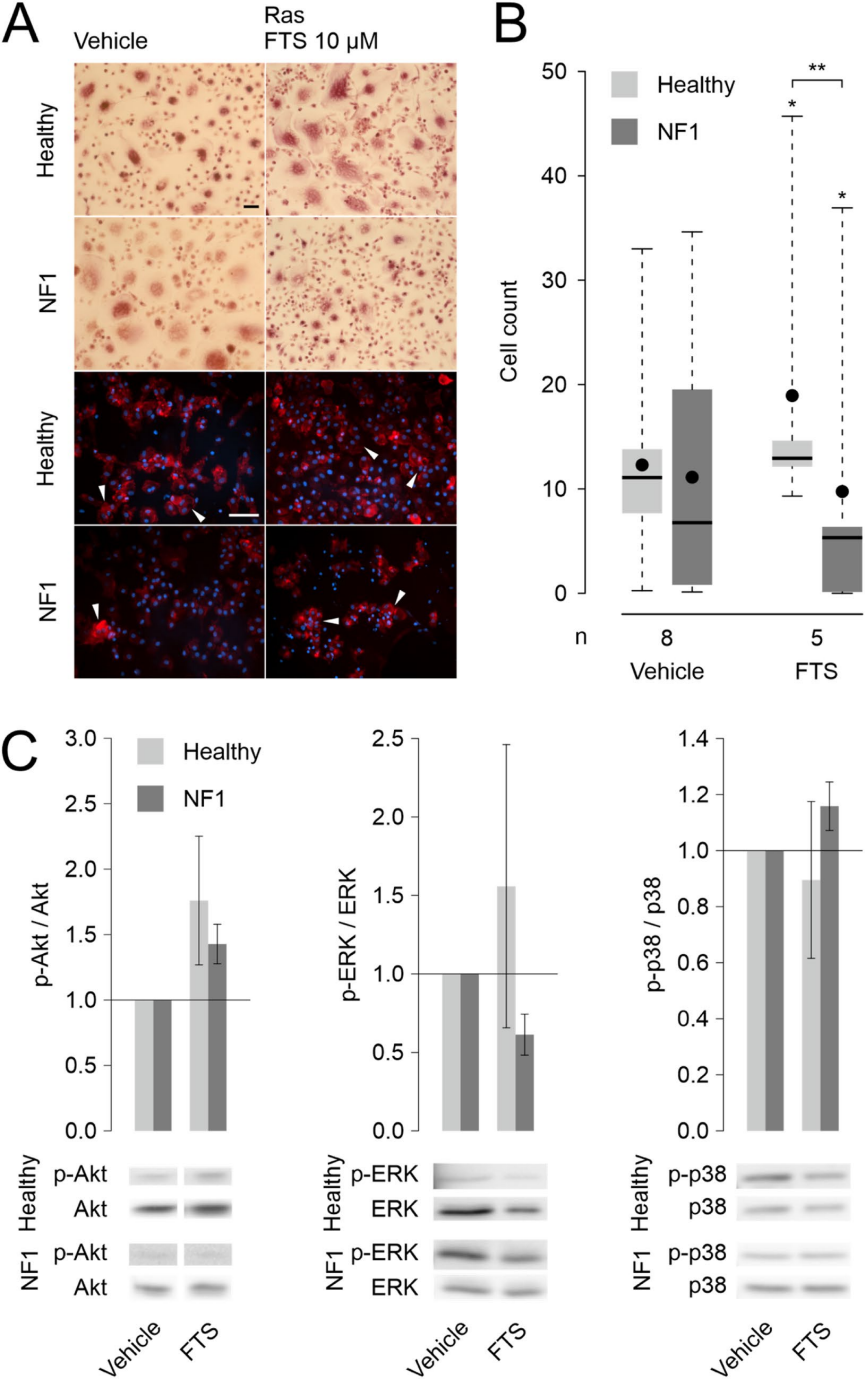
The effect of inhibition of Ras on osteoclast differentiation. (**a**) The first two rows show the tartrate-resistant acid phosphatase staining of cells isolated from the peripheral blood of control persons and patients with neurofibromatosis type 1 (NF1) and cultured on glass with the Ras inhibitor farnesyl thiosalicylic acid (FTS). The two bottom rows show actin staining (red) on bone together with nuclei (blue). White arrows indicate examples of osteoclasts in the fluorescence images. Scale bars are 50 μm. (**b**) Counts of osteoclasts after 4–5 days of differentiation in the presence of FTS. The thick horizontal line represents the median, the black dot shows the mean, the box displays the 1st and 3rd quartiles and the whiskers show the range of values. The number (n) of matched healthy-NF1 pairs is shown for each treatment. Asterisks denote significance of difference to vehicle control (above each box) or between healthy and NF1. *, 0.01 < *P* < 0.05; **, 0.001 < *P* < 0.01; ***, *P* < 0.001. (**c**) Effects of FTS on the phosphorylation of Akt (60 kDa), ERK1/2 (42/44 kDa), and p38 (41 kDa). The bars show mean ± standard deviation of phosphorylated protein / total protein normalized relative to the vehicle treatment. The data are representative of three experiments with cells from different individuals. Examples of bands observed by western blotting are shown below each figure. Since the samples shown in Figs. 2, 3 and 4 were run on the same gel, the vehicle control band for each antibody is shared by the figures. (Color figure online)

The number of osteoclasts increased, when cells from control persons were treated with the Ras inhibitor FTS (Fig. 4). Interestingly, when NF1 cells, where Ras is presumed to be over-activated, were treated with the same inhibitor, the number of osteoclasts was significantly lower than the number of osteoclasts from control persons (Fig. 4a and b). The western blot results showed that this finding correlated with the phosphorylation of ERK1/2, as FTS inhibited activation of ERK1/2 in NF1 cells (Fig. 4c). In addition, the inhibition of Ras increased the phosphorylation of Akt in both healthy and NF1 cells (Fig. 4c, left panel).

## Discussion

Most previous studies related to osteoclast signaling pathways have been performed using rodent cells. Thus, the current data extend our understanding on the signaling involved in human osteoclast differentiation and function, even though further mechanistic studies are required to fully understand the present observations. In all conditions, viable osteoclasts were observed. Inhibition of PKC, MEK, PI3K, and mTOR decreased the number of human osteoclasts, which corroborates the earlier studies carried out with mouse osteoclasts [13, 16–18, 20].

One of our major findings was that the inhibition of p38 promoted differentiation of human peripheral blood monocytes into osteoclasts in samples derived from control persons and NF1 patients. This finding is contradictory to the observations in rodent studies where p38 inhibition suppressed osteoclast differentiation [6, 7]. In our study, blocking p38 resulted in osteoclasts that were larger in size and that actively formed F-actin ring, while no increase in the resorption capacity was observed as estimated by CTX assay of SB203580 treated cells. Unfortunately, the CTX assay was not performed in BIRB796 treated cultures. Our results are in line with Li et al. (2002) [6], who showed that p38 regulates mouse osteoclastogenesis by affecting especially osteoclast precursor differentiation into osteoclasts but not the resorption activity.

The inhibition of p38 increased the phosphorylation of ERK1/2 in healthy cells but not in NF1 cells. It appears that there is a strong interaction between the p38 and Ras/Raf/MEK/ERK1/2 pathways. Combined inhibition of p38 and MEK counteracted the osteoclastogenesis-promoting effect of p38 inhibition alone. This further corroborates the conclusion that p38 interacts with the Ras/Raf/MEK/ERK1/2 pathway in osteoclast differentiation. Since dose-response analysis of p38 inhibition was outside the scope of the present study, we cannot fully exclude off-target effects of the p38 inhibitors. However, both SB203580 and BIRB796 are relatively specific to p38 [38], and their concordant effects on osteoclastogenesis further support the conclusion that the observed effects are indeed related to p38 inhibition. The concentrations of SB203580 and BIRB796 used in the present study have previously been used in several studies of other cell types [6–8, 39–41].

Another major finding of our study was that the number of osteoclasts appeared to correlate with the phosphorylation of ERK1/2. Different responses to Ras inhibition with FTS in cells from NF1 and control samples were observed; FTS increased osteoclastogenesis and ERK1/2 phosphorylation of control samples and decreased osteoclastogenesis and ERK1/2 phosphorylation in NF1 samples. A similar association was observed also in our other experiments showing that p38 inhibition increases ERK1/2 phosphorylation, a correlation previously observed in another cell type [39], and osteoclastogenesis in control samples. Inhibitors of MEK, PKC, mTOR, and PI3K decreased ERK1/2 phosphorylation in NF1 samples and reduced markedly osteoclast numbers. Even though the effect on ERK1/2 phosphorylation was not observed in samples from control persons, the inhibition of PKC and MEK induced a significant decrease in osteoclast numbers. In addition to the role of ERK1/2, the results inform how the regulation of Ras by, e.g., neurofibromin modulates the effects of inhibiting the downstream signaling. ERK1/2 phosphorylation might also be dose-dependent.

The inhibition of PI3K in healthy cells did not have a significant effect on the number of osteoclasts, but the formation of F-actin rings was disrupted. This is in accordance with earlier results in mice, which have shown that the inhibition of PI3K interferes with the formation of F-actin structure and osteoclast attachment on the bone [42]. In the current study, the inhibition of PI3K had little effect on the phosphorylation of ERK1/2, Akt, or p38 in healthy cells. This suggests that the PI3K protein has an independent role in F-actin ring formation. Moreover, healthy spindle shaped cells were seen, suggesting that the inhibitor treatment affected specifically osteoclast differentiation and not the viability of the mononuclear cells.

In conclusion, the previously suggested effects of PKC, MEK, PI3K, and mTOR inhibition were confirmed in human osteoclasts, but not in the case of p38, where inhibition of p38 resulted in increased osteoclastogenesis. The increase in osteoclastogenesis induced by p38 inhibition seemed to associate with increased ERK1/2 phosphorylation. Multiple observations in the current study support the speculation that ERK1/2 phosphorylation may act as a key driver of human osteoclast differentiation. These results highlight the need for a deeper understanding of molecular mechanisms and cellular events that affect human osteoclastogenesis.

## Data Availability

All data are included in the manuscript.

## Abbreviations

CTX: Carboxy-terminal cross-linking telopeptide of type I collagen
ERK1/2: Extracellular-signal-regulated kinase
M-CSF: Macrophage colony-stimulating factor
MEK: Mitogen-activated protein kinase
mTOR: Mammalian target of rapamycin, mechanistic target of rapamycin, sirolimus
NF1: Neurofibromatosis type 1
*NF1*: Human *NF1* gene
NIH: National Institutes of Health
PKC: Protein kinase C
PI3K: Phosphoinositide 3-kinase
RANKL: Receptor activator of nuclear factor kappa-B ligand
TRACP: Tartrate-resistant acid phosphatase 5B
